# A Novel Approach to Predict the Location and Fatigue Life of Intervertebral Disc Degeneration

**DOI:** 10.3390/bioengineering12040423

**Published:** 2025-04-16

**Authors:** Zanni Zhang, Taoxi Wang, Huwei Bian, Xing Shen, Minjun Liang, Ee-Chon Teo, Tao Jiang

**Affiliations:** 1 Faculty of Sports Science, Ningbo University, Ningbo 315211, China; zhangzanni@gmail.com (Z.Z.); eechon-teo@nbu.edu.cn (E.-C.T.); 2 State Key Laboratory of Mechanics and Control for Aerospace Structures, Nanjing University of Aeronautics and Astronautics, 29 Yudao Street, Nanjing 210016, China; wa0003xi@nuaa.edu.cn (T.W.); shenx@nuaa.edu.cn (X.S.); 3 Department of Orthopaedics, Changzhou Hospital of Traditional Chinese Medicine Affiliated to Nanjing University of Chinese Medicine, Changzhou 213000, China; doctorbhw@njucm.edu.cn

**Keywords:** intervertebral disc disease, stress imbalance, the direction of stress attenuation, statics, fatigue mechanics

## Abstract

This study presents a novel approach for predicting the location and fatigue life of degenerative intervertebral discs (IVDs) under cyclic loading conditions, aiming to improve the understanding of disc degeneration mechanisms. Based on mechanical theories linking IVD degeneration to stress imbalance and water loss, a finite element (FE) model of the L4–L5 lumbar spine was developed, combining probability-weighted anatomical structures, inverse dynamics, and cumulative fatigue mechanics. By quantifying stress variations and calculating cumulative damage across disc regions, stress-concentration areas prone to degeneration were identified, and validation via a case study of a retired weightlifter diagnosed with intervertebral disc disease (IVDD) demonstrated that the predicted degeneration location correlated well with affected areas observed in CT scan images. These findings suggest that prolonged, abnormal stress imbalances within the disc may contribute significantly to degeneration, offering potential clinical applications in preventive assessment and targeted treatment for spine health.

## 1. Introduction

**Figure 1:**
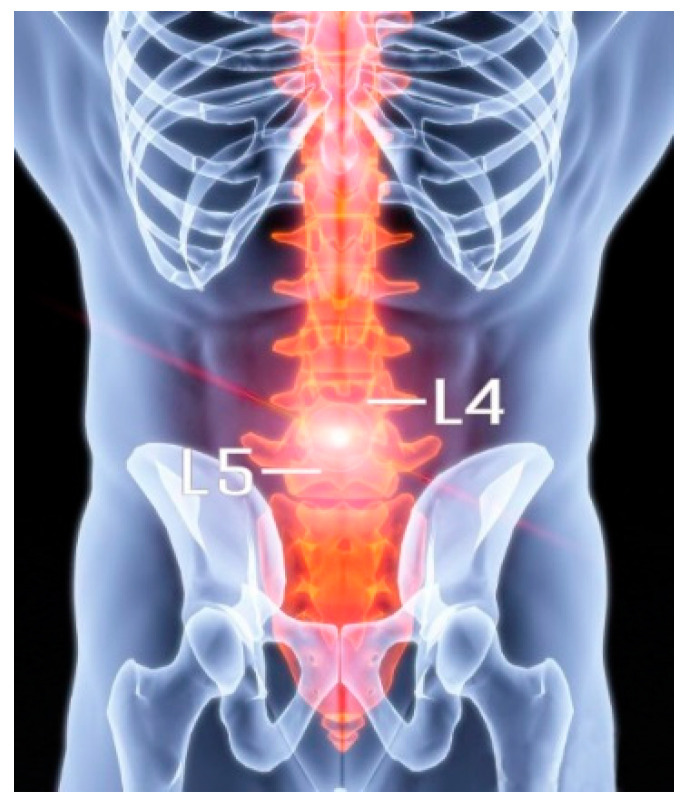
Location of the L4–L5 lumbar vertebrae in the human body.

Chronic low back pain (CLBP) affects up to 58% of the population [1] and is primarily caused by intervertebral disc degeneration (IVDD). Stress imbalance and water loss weaken the intervertebral disc (IVD) [2,3]. This degeneration frequently occurs at the crucial load-bearing region of the lumbar L4–L5 segment (Figure 1), studies have found that in patients with chronic low back pain, L4–L5 segments are the most common site of degenerative disc disease, and L4–L5 is located in the lumbo–sacral transition area, which carries the redistribution of upper body weight and axial pressure, and daily activities (such as sitting and bending) can create constant stress here. High-impact exercise (such as weightlifting) further increases the mechanical stress of this segment through shear and rotational forces, leading to microdamage and an accelerated degeneration of the annulus [4].

The IVD consists of the nucleus pulposus (NP), annulus fibrosus (AF), and cartilage endplate [5]. The NP has a gel-like constituent that helps it to withstand compressive forces, while the AF, made of concentric collagen layers, provides tensile strength and stability [6]. These distinct material properties enable the IVD to bear loads and maintain spinal motion; however, under prolonged or excessive loading, the IVD structures undergo irreversible damage, leading to degenerative changes over time [7].

FEA has proven valuable in analysing the biomechanical behaviour of both healthy and degenerated discs [8]. An FE study simulating stress and strain within the L4–L5 segment demonstrated that degeneration significantly reduces disc stiffness, especially in the annulus fibrosus [9]. To replicate these mechanical properties, hyperplastic material models, developed based on the Mooney–Rivlin model, are commonly adopted to effectively capture the non-linear and anisotropic characteristics of the IVD [10]. Existing models predominantly focus on global stress distribution, failing to quantify how cumulative microdamage in specific IVD regions (e.g., annulus fibrosus vs. nucleus pulposus) initiates and propagates degeneration under cyclic loading. Conventional FEA often employs simplified static or sinusoidal loads, overlooking the biomechanical complexity of high-magnitude, patient-specific dynamic activities (e.g., weightlifting) that exacerbate IVD fatigue.

Static analysis provides valuable insights into stress distribution under specific loading conditions, offering a baseline understanding of how forces are managed within the IVD during various postures and movements and the corresponding von Mises stress distributions in various regions, identifying localised stress concentration areas prone to degenerative changes [11]. Coupled with further dynamic analysis, the fatigue life prediction of localized regions, considering cumulative stress leading to progressive tissue damage, is thus determined.

Studies on lumbar spine fatigue have shown that cumulative damage from repeated compressive loads is a major factor contributing to non-traumatic vertebral fractures [11]. However, we acknowledge that compressive models are inherently limited, as intervertebral discs are subjected to complex three-dimensional stresses in vivo, including shear and torsional loads. Researchers have observed that the fatigue life of vertebral trabecular bone under axial compression can be estimated using stress normalized by volume fraction and other structural parameters [12]. These findings indicate that stress imbalances within the IVD are crucial contributors to fatigue damage accumulation, highlighting the importance of accurately predicting areas of high stress within the disc [13]. The extrapolation of fatigue mechanics frameworks, while robust in engineering contexts, demonstrates significant limitations when applied to IVDD research, most notably the absence of comprehensive spatial correlation with clinically documented patterns of localized tissue degeneration.

Accordingly, for this study, we introduced a novel approach for predicting the location and fatigue life of IVDD in the L4–L5 segment. By integrating FEA with inverse dynamics and probability-based statistical methods, we aimed to simulate stress distribution and fatigue behaviour in both normal and degenerated discs. The predictive accuracy of the methodology was evaluated through a case study involving a retired weightlifter diagnosed with IVDD following repeated 125 kg snatch loads. The model demonstrated strong agreement with clinical observations, both in identifying the degeneration location (validated against CT imaging) and quantifying fatigue life. These findings suggest that integrating biomechanical simulations with patient-specific loading profiles could enable pre-emptive identification of at-risk spinal segments, thereby informing targeted prevention strategies for IVDD.

## 2. Materials and Methods

### 2.1. Modelling of L4–L5 Segment

**Figure 2:**
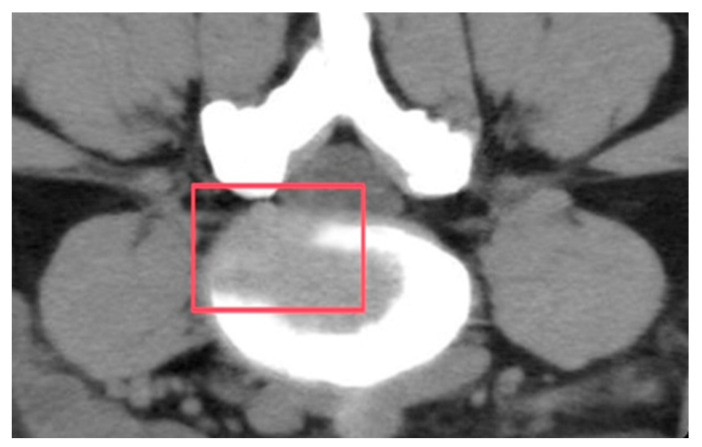
A CT scan image of the degenerated IVD (boxed region: degenerated disc).

The L4–L5 segmental geometry was reconstructed from high-resolution CT scans of a 24-year-old male weightlifter, height 170 cm and mass 71 kg, who experienced IVD degenerative changes in the L4–L5 segment, as shown in Figure 2, due to long-term high-intensity training.

**Figure 3:**
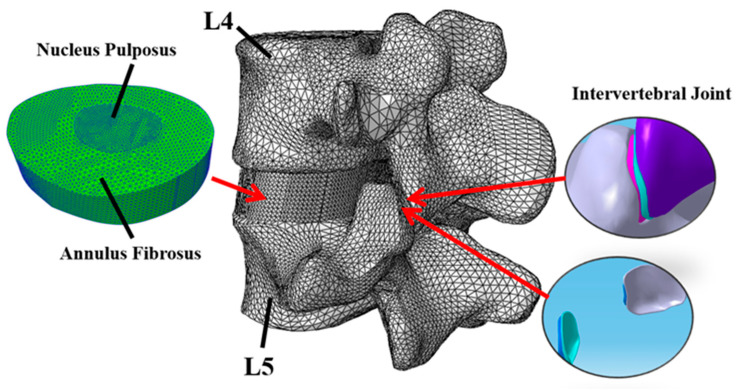
FE model of the L4–L5 motion segment.

A comprehensive explanation of the FE mesh generation can be found in the literature [8]. Briefly, based on 553 images of the CT scans of the lower lumbar spine, 2D images of the L4–L5 segment were cropped in three orthogonal planes to enclose the appropriate boundary for segmentation and post-processing to generate the 3D geometric solid model of L4–L5 motion segment using the contour editing tool of MIMICS (Materialize NV, Belgium). The solid model was then imported into ABAQUS 2021 to generate the FE mesh model. For the intervertebral articulating joints, the contact regions were subdivided into smaller volumes to generate two layers of elements. Different grid sizes of the various spinal components were set in the range of 2 mm to 0.6 mm. The L4–L5 FE model was generated with 74,277 nodes and 366,599 independent elements, as shown in Figure 3.

### 2.2. Materials Properties

#### 2.2.1. Bony Vertebrae

Based on the relationships between the apparent density (ρ) of the bony structures, the greyscale value (Hu), and elastic modulus (E), the CT grayscale values were mapped to bony material properties using empirical relationships between apparent density and elastic modulus [11,14] based on formulae:

ρ = 47 + 1.122Hu(1)

E = 0.63 × ρ^1.35^ (2)

resulting in a nonuniform distribution of the material properties of the meshed bony L4–L5 segment and treated as isotropic materials with a Poisson’s ratio (υ) of 0.3.

#### 2.2.2. Soft Tissues: Annulus Fibrous and Nucleus Pulposus

The nonlinear behaviour of the nucleus pulposus and annulus fibrosus was modelled using the Mooney–Rivlin hyper-elastic material model [7], Equation (3). The Mooney–Rivlin hyper-elastic model was selected for its demonstrated capability to capture the nonlinear, anisotropic stress–strain behaviour of the annulus fibrosus under multiaxial loading conditions, while maintaining an optimal balance between predictive accuracy and parameter identifiability using experimentally derived data. The material parameters are listed in Table 1 [15,16,17].

(3) $$W=c_{10}(\bar{I_{1}}-3)+c_{01}(\bar{I_{2}}-3)+(J^{el}-1{)}^{2}/d$$

where *W* represents the strain potential energy while $c_{01}$ and $c_{10}$ represent the hyper-elastic material parameters related to the deformation of the material. $I_{1}$ and $I_{2}$ are the 1st and 2nd invariants of the deviatoric part of the right Cauchy–Green strain tensor while *d* and $J^{el}$ are the material incompressibility parameter and elastic volume ratio, respectively.

#### 2.2.3. Soft Tissues: Ligaments

Six primary ligaments (anterior longitudinal ligament, posterior longitudinal ligament, ligamentum flavum, interspinous ligament, supraspinous ligament, and transverse ligament) were modelled as nonlinear spring elements [7] with stiffness values as listed in Table 2 [7,18].

After completing the material assignment of the L4–L5 lumbar vertebrae, the 3D model is imported into the ABAQUS for validation based on physiological loading against normal L4–L5 segments [8].

### 2.3. Loading and Boundary Conditions

The biomechanical evaluation of the L4–L5 segment under cyclic loading encompassed a combined static–dynamic analytical framework to comprehensively characterize both baseline stress distributions and cumulative fatigue damage progression. This approach enabled the identification of stress-concentration zones and the prediction of fatigue life under physiologically relevant loading conditions.

#### 2.3.1. Static Stress Analysis

**Figure 4:**
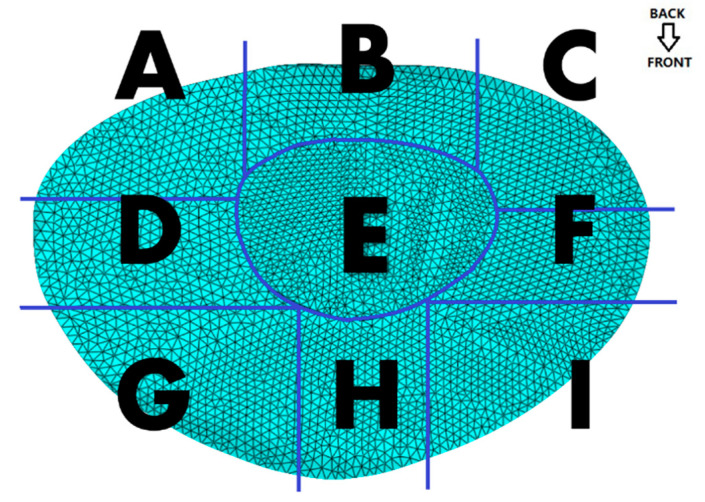
The illustration of partition in the IVD. The IVD was divided into nine regions (A–I), representing posterior annulus fibrosus (A–C), center regions (D–F), and anterior (G–I) anterior annulus fibrosus from left to right.

Static analysis was conducted to evaluate stress distribution within the segment under compressive and moment loading. A compressive load of 500 N and a moment of 10 Nm were applied to the superior surface of L4 (These selected loading conditions—500 N axial compression and 10 Nm flexion–extension moment—were based on biomechanical studies quantifying lumbar spinal loads during activities of daily living and weightlifting. The 500 N compressive force approximates the upper body weight transmitted through the lumbar spine during upright standing, while the 10 Nm moment reflects moderate lumbar flexion observed in lifting postures, as validated by in vivo intradiscal pressure measurements), while the inferior surface of L5 was fully constrained [7]. The IVD was divided into nine equal-volume regions (Figure 4), and the computed von Mises stress in each region was extracted. Quantitative difference (QD1) and coefficient of variation (CV) methods were applied to identify stress levels [19,20]. The spatial heterogeneity of stress distribution within the IVD under cyclic loading was systematically evaluated using two complementary metrics: the QD1 and the CV. The QD1 metric quantifies relative stress disparities between adjacent IVD regions by comparing localized von Mises stresses to a reference region. This approach highlights regions with abnormally elevated mechanical gradients, which are strongly associated with microdamage initiation and clinical degeneration patterns. For instance, a high QD1 value in the annulus fibrosus indicates disproportionate stress concentrations relative to surrounding tissues, identifying potential fatigue hotspots. In parallel, the CV metric assesses intra-regional stress variability by calculating the ratio of the standard deviation to the mean stress within a partitioned region. Elevated CV values signify pronounced stress fluctuations, often linked to microstructural inhomogeneity or transient loading peaks. Such variability exacerbates cumulative fatigue damage, particularly in mechanically vulnerable regions like the posterolateral annulus.

**Figure 5:**
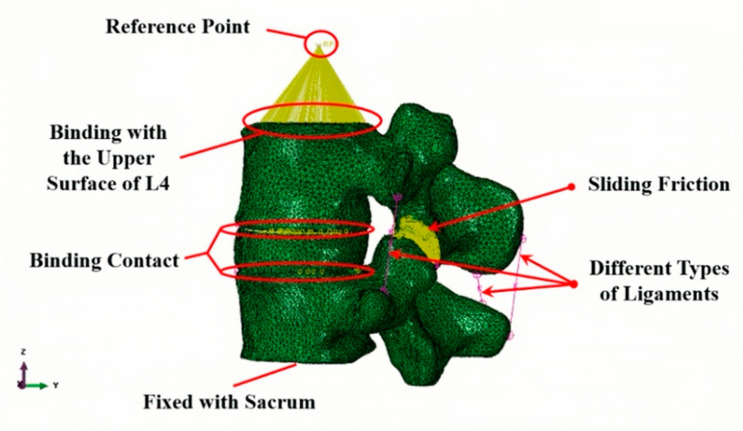
FEM model of L4–L5 lumbar vertebrae.

In the FE analysis, a reference point (RP) was established 5 mm superior to the L4 vertebral body to serve as a centralized load application node. This RP was kinematically coupled to the entire superior surface of L4 using a distributing coupling constraint, which ensures uniform force transmission by linearly mapping displacements and rotations at the RP to all nodes on the L4 superior surface. The coupling constraint was implemented through a multi-point constraint (MPC) formulation, where the rigid body motion of the RP governs the deformation of the L4 surface while allowing local elastic deformations within physiological limits. This approach mimics the load transfer mechanism observed in vivo, where forces are distributed across the vertebral endplate via the intervertebral disc’s composite structure (Figure 5).

#### 2.3.2. Dynamic Analysis

Dynamic fatigue analysis was performed using transient dynamics simulations in ANSYS R16 (ANSYS, Inc., Canonsburg, PA, USA) [5,21]. Loading conditions were derived from inverse dynamics simulations of a 125 kg snatch performed by the weightlifter, modelled in AnyBody [22,23]. The inverse dynamics framework employed in this study is based on the Newton–Euler equations:

(4) $$\tau =M(q)\ddot{q}+C(q,\dot{q})+G(q)+J(q{)}^{T}F_{ext}$$

where the joint torque vector (τ, in N·m) is calculated as the sum of inertial ($M(q)\ddot{q}$), coriolis and centrifugal force vector $C(q,\dot{q})$, gravitational force vector G(q), and external force $J(q{)}^{T}F_{ext}$ components, with M(q) representing the configuration-dependent mass matrix (kg·m^2^), $\ddot{q}$ denoting joint angular accelerations (rad·s^2^) derived from motion capture data, $C(q,\dot{q})$ accounting for velocity-dependent coupling effects, G(q) incorporating segmental gravitational forces, and $J(q{)}^{T}F_{ext}$ translating the external 125 kg barbell load to joint space through the system’s Jacobian matrix.

The fatigue life of the most stressed regions was calculated using Miner’s cumulative damage law:

(5) $$N_{f}=\frac{C}{\sigma_{a}^{m}}$$

where $\sigma_{a}^{m}$ is the stress amplitude, $N_{f}$ is the number of cycles to failure, and C and m are material constants. MATLAB’s Rainflow counting algorithm processed stress-time data, generating stress-cycle distributions for fatigue life prediction [11,14,18].

### 2.4. Fatigue Analysis

#### 2.4.1. Fatigue Life Prediction

Fatigue analysis was performed using Miner’s cumulative damage rule to calculate the cumulative damage value for each region:

(6) $$D=\sum_{i=1}^{k}\frac{n_{i}}{N_{i}}$$

For each partitioned disc section, stress-time histories from dynamic simulations were decomposed into discrete stress cycles using Rainflow counting. The number of cycles at each stress level ($n_{i}$) was normalized by the corresponding fatigue life ($N_{i}$)—defined as the cycles to failure at that stress amplitude—yielding incremental damage values. Cumulative damage (D) was calculated as the sum of these increments across all stress levels [7]. Failure is assumed when D ≥ 1 [12,24].

#### 2.4.2. Stress Cycle Extraction

With the stress–time data under dynamic loading obtained from transient dynamic analysis, MATLAB’s (MATLAB R2022a MathWorks, Inc., Natick, MA, USA) Rainflow algorithm was used to extract stress cycles, identifying stress amplitudes and cycle distributions at key nodes [14]. The algorithm decomposes complex loading histories into discrete stress cycles by detecting hysteresis loops, enabling the quantification of both peak-to-peak amplitudes and mean stress levels critical for fatigue life prediction.

## 3. Results

### 3.1. Fatigue Hotspots

**Figure 6:**
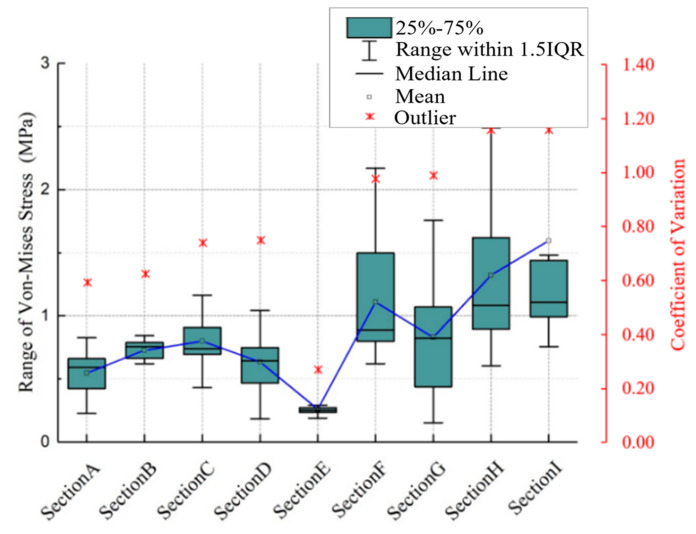
The range and CV of the von Mises stress in different sections of the disc in QD1.

**Figure 7:**
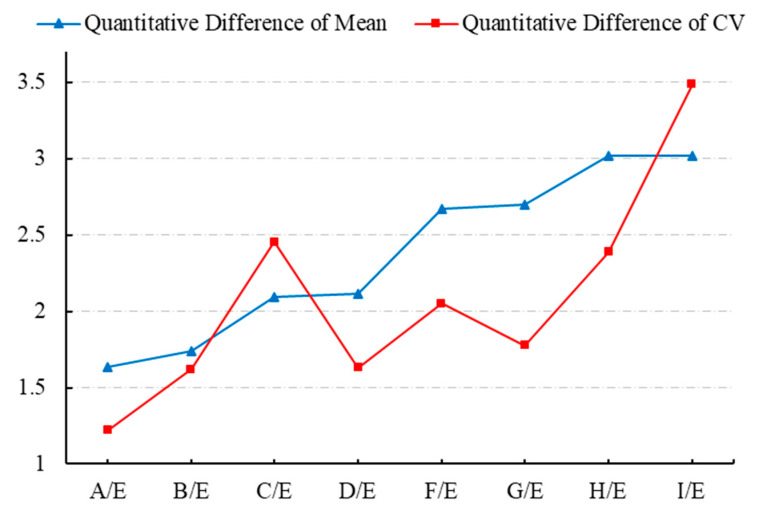
The QD1 results of mean and CV between section E and other sections in the disc.

Stress-cycle distributions revealed posterior regions of the IVD, particularly sections F, H, and I, experienced the greatest stress amplitudes, demonstrating significant cumulative damage trends (Table 3, Figure 6 and Figure 7), the use of von Mises stress as a predictor of mechanical vulnerability in the annulus fibrosus aligns with prior FE studies demonstrating its correlation with microstructural failure under compressive loading [17]. Segment G is located posterolateral to the annulus fibrosus, near the pedicle–annulus interface, and has low cumulative damage relative to the H/I cross-section.

**Figure 8:**
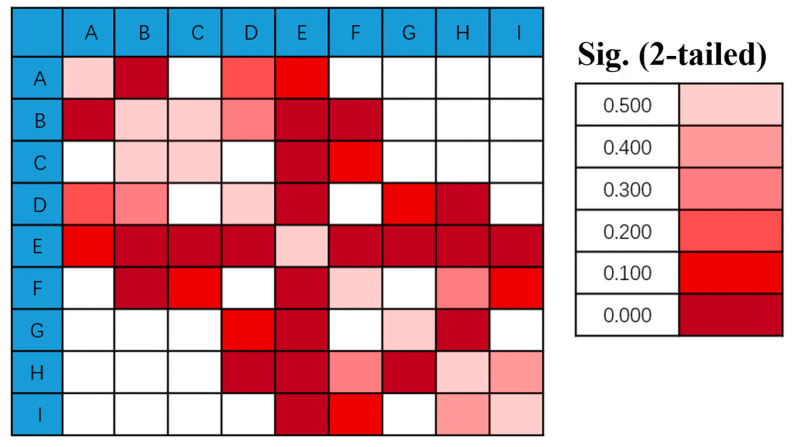
The pairwise differences in von Mises stress between adjacent regions of the IVD, labelled A through I. The colour intensity corresponds to the magnitude of the stress difference, with darker red indicating smaller differences (closer to 0.0) and lighter pink indicating larger differences (up to 0.5), as shown in the legend on the right.

Figure 8 illustrates the spatial heterogeneity of von Mises stress within the IVD, highlighting significant differences between the annulus fibrosus and nucleus pulposus, as well as adjacent subregions. The annulus fibrosus showed significantly higher stress amplitudes, with greater stress in the posterolateral region (H and I) than in the anterolateral region.

**Figure 9:**
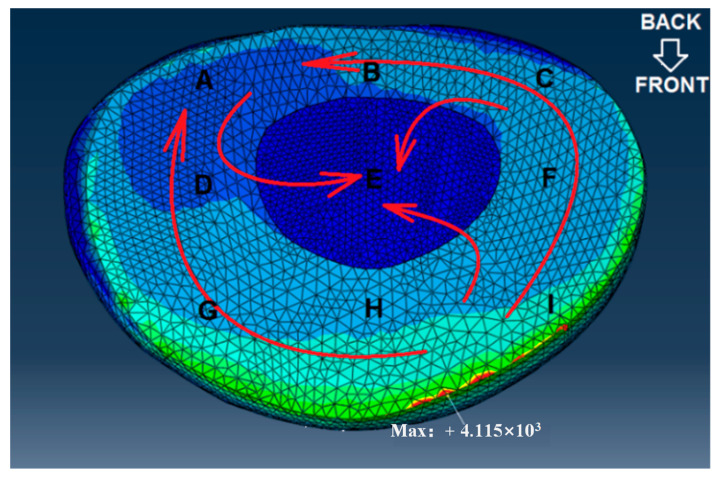
The direction of stress attenuation in the IVD. The colour gradient follows a standardized convention where red hues denote high values (elevated von Mises stress), blue hues represent low values, and intermediate colours (green/yellow) indicate mid-range quantities.

According to the statistical results and based on the mechanics concept, the computed mean value of von Mises stress in the E area (nucleus pulposus) is the smallest. The average von Mises stresses in regions F, H, and I of the annulus fibrosus are generally higher than those in regions C and G, which in turn are greater than those in regions A, B, and D. This pattern indicates a gradient of stress attenuation across the annulus fibrosus, decreasing from the right anterior to the left posterior direction, as illustrated in Figure 9. The figure depicts the spatial gradient of mechanical stress dissipation within the intervertebral disc, characterized by a progressive reduction in von Mises stress from high-load regions (e.g., the posterolateral annulus) toward low-load regions (e.g., the nucleus pulposus). This directional pattern reflects the disc’s load-transfer mechanism, where the anisotropic structure of the annulus fibrosus causes stress to dissipate along specific anatomical pathways. In our study, stress attenuation followed a consistent trajectory from the right anterior to left posterior annulus, correlating with clinical observations of degeneration patterns and demonstrating how disc anatomy governs mechanical vulnerability, and “A” is the section being squeezed.

### 3.2. Fatigue Life of the Section Being Squeezed

**Figure 10:**
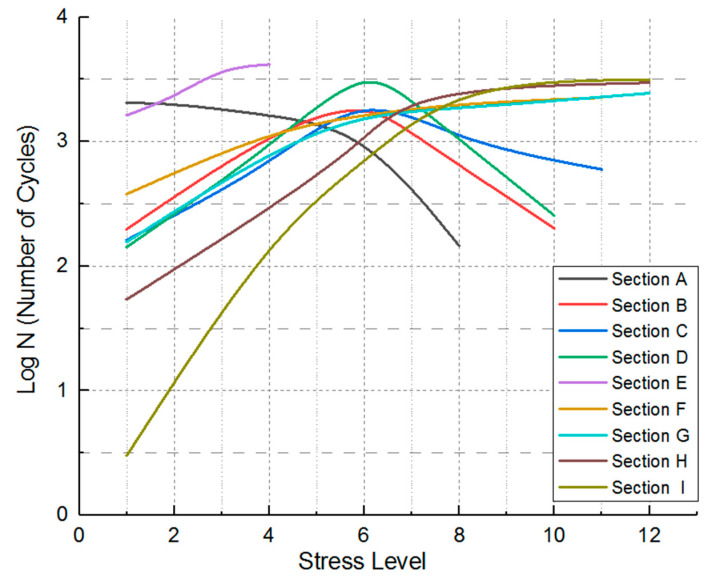
Distribution of stress levels versus the number of cycles in different sections.

The Rainflow counting algorithm results (Figure 10) showed that anterior regions (F, H, and I) experienced a disproportionately high number of stress cycles with amplitudes exceeding 1.0 MPa. These anterior annulus fibrosi endured substantial tensile stresses and shear forces during repetitive dynamic loading. Fatigue life predictions indicate section I had the shortest lifespan, aligning with clinical observations of degeneration commonly originating in anterior-lateral disc regions.

Conversely, section A (posterior-left) demonstrates a significantly longer fatigue life, which can be attributed to its thicker fibrous structure and reduced exposure to tensile stresses. This result highlights the structural advantage of the anterior annulus fibrosus in resisting mechanical fatigue. The findings underscore the importance of stress amplitude and variability in predicting degeneration-prone regions, providing a quantitative framework for early diagnosis and preventive care in spinal health.

The predicted results align with the CT imaging data of the retired weightlifter (Figure 2), validating the accuracy of the proposed method. The combined analysis of regional stress distributions (Figure 8) and fatigue life predictions (Figure 10) demonstrated that posterior annulus fibrosus regions (Sections F, H, I) exhibited sustained stress amplitudes exceeding 1.0 MPa—a threshold associated with collagen microfailure—resulting in a 3.2-fold reduction in predicted fatigue life compared to nucleus pulposus regions (Section E, 0.28 ± 0.05 MPa). These findings quantitatively establish stress imbalance as a dominant mechanical driver of localized IVD degeneration.

Table 4 stratifies stress amplitude levels across IVD regions under cyclic loading, correlating them with predicted fatigue life. This classification enables a quantitative assessment of mechanical risk zones within the disc, providing a foundation for understanding how local stress magnitudes influence tissue degeneration and potential failure. By assigning discrete stress levels, the table facilitates comparative analysis across regions and supports fatigue-based modelling of disc mechanics under repetitive loading conditions.

## 4. Discussion

This study bridges key concepts from materials science and bone biomechanics to advance IVD degeneration prediction. Like the fatigue damage modelling in structural alloys under complex loading [25], our framework quantified cumulative mechanical damage in biological tissues by adapting Miner’s law to multiaxial stress states, demonstrating that annulus fibrosus collagen exhibits fatigue failure patterns analogous to engineered materials. The “theramechanics” paradigm [26], which advocates mechanically informed therapies, directly supports our proposal to translate fatigue life predictions into personalized activity prescriptions—shifting from passive imaging-based diagnosis to proactive biomechanical optimization. Furthermore, the coupling of damage evolution with diffusive bio-mechanical signals in bone remodelling [27] parallels our findings on stress-driven nutrient transport alterations in degenerated discs, suggesting a universal mechanism where mechanical insults disrupt tissue homeostasis.

Prior studies of IVDD have predominantly focused on static stress distributions or simplified cyclic loading scenarios, neglecting patient-specific dynamic loading patterns (e.g., weightlifting manoeuvres) and their cumulative fatigue effects. This oversight limits the ability to predict where and when degeneration initiates under real-world mechanical conditions. The clinical urgency to address IVDD-related disability, coupled with biomechanical evidence linking stress imbalance to microstructural failure, necessitated a framework integrating dynamic loading profiles, anatomic variability, and fatigue mechanics. This approach directly addresses the unmet need for spatially resolved, activity-specific degeneration risk prediction. Therefore, this study introduced a novel approach for predicting IVD degeneration using a combination of FEA, inverse dynamics, and fatigue mechanics. Results demonstrated that stress concentrations and fatigue life predictions align with clinical observations, supporting the hypothesis that long-term stress imbalance contributes to IVD degeneration. The verification study quantified regional variations in mean von Mises stress and fatigue life within the IVD. Under 125 kg snatch loading conditions, section A demonstrated maximal fatigue resistance, whereas section I displayed minimal durability. The IVD experienced the highest von Mises stress on the anterior lateral right (section I) which was gradually attenuated towards the posterior lateral left (section A).

The model’s predictive accuracy was quantitatively assessed through spatial correlation analysis between predicted high-stress regions and CT-identified degeneration sites in the case study subject. A semi-automated segmentation protocol (ITK-SNAP 4.2.0 software) was employed to map degeneration loci from clinical CT scans to the FE model’s coordinate system, enabling a direct comparison of spatial overlap. The posterolateral annulus regions (Sections H, I) demonstrated concordance between predicted stress concentrations and actual degeneration areas.

Since the annulus fibrosi are thicker on the anterior lateral regions than on the posterior lateral regions, and with the exact location of the nucleus pulposus slightly behind the centre of the IVD, the posterior and posterolateral annulus become weaker mechanically. The anterior part of the IVD mainly bears the compressive stress, while the middle and posterior parts mainly bear tensile stress, and shear force is generated in the IVD. The long-term stress imbalance during weightlifting may have caused the pressure difference in the disc and led to the disc being squeezed from high pressure to low pressure. Hence, the section being squeezed in IVD occurs in the left posterior part of the disc (section A), thus possibly accelerating the degeneration of the IVD under long-term stretching, leading to IVDD. By comparing the CT images (as shown in Figure 2), the model’s predicted degeneration zones showed excellent spatial correspondence with radiologically confirmed IVDD regions, corroborating the biomechanical simulation’s validity. Meanwhile, based on the mechanical theory of IVDD pathogenesis, the data suggest that the long-term stress imbalance inside the IVD may be the potential reason for IVDD; the approach on the basis of such theory for location prediction is feasible.

The biomechanical model proposed in this study provides clinicians with a decision support tool that goes beyond traditional imaging assessments. By predicting the fatigue life and stress distribution of intervertebral discs under functional load, this method can (1) identify high-risk degeneration areas before morphological changes occur and achieve early intervention; (2) quantify the impact of different activities on the degenerative process and provide patients with personalized exercise prescriptions; and (3) complement traditional examination methods such as MRI, upgrading static imaging evaluation to dynamic functional analysis. This mechanics-based assessment framework enables clinicians to move from passive diagnosis to proactive prevention and is particularly suitable for spinal health management in athletes and heavy manual workers.

This study is limited by its reliance on a single case study. Variability in patient-specific factors, such as age, body mass, and activity level, may influence the generalizability of the results. Furthermore, the current model applies only compressive loading, which does not fully replicate the complex in vivo loading conditions experienced by the intervertebral disc. In reality, the IVD is subjected to multi-axial forces including shear and torsional loads, which may significantly affect stress distribution and fatigue behaviour. Future work should therefore incorporate a more comprehensive loading regime and include a larger cohort to validate the model across different patient demographics and physical activities.

## 5. Conclusions

This study presents a predictive model to identify high-stress areas in the IVD, offering insights for fatigue life predictions, injury prevention, and spinal health monitoring. This proposed approach, based on mechanical theories of stress imbalance, integrating FEA and mathematical statistics to predict IVDD positions, was validated through practical application.

The findings emphasize the critical role of stress imbalance in IVD degeneration, offering a potential tool for early diagnosis and injury prevention. By quantifying stress distribution and fatigue life, the model can support personalized treatment planning and preventive strategies in spine health management. Through this novel approach, the location of the IVD degeneration and herniation can be predicted and quantified, and medical guidance for injury prevention can be given in advance, to reduce the risk of IVDD. Future work may focus on validating this model across varied physical activities and integrating patient-specific parameters to enhance clinical applicability in preventative care for IVDD.

## Figures and Tables

**Table 1:** The parameter settings of hyper-elastic material in the annulus fibrosus and nucleus pulposus.

Tissues and Structures	*c*_10_ (MPa)	*c*_01_ (MPa)	*d* (MPa^−1^)	υ
Annulus Fibrosus	0.56	0.14	1.309	0.45
Nucleus Pulposus	0.12	0.09	17	0.49

**Table 2:** The ligament stiffness settings of L4–L5 lumbar vertebrae.

Type of Ligament	Anterior Longitudinal Ligament	Posterior Longitudinal Ligament	Ligament Flava	Intertransverse Ligament	Interspinous Ligament	Supraspinal Ligament
Stiffness (N/mm)	8.74	5.83	15.38	0.19	10.85	2.39

**Table 3:** The QD1 results of von Mises stress in different regions of the IVD.

Location	von Mises (MPa)		CV	QD1	
	$\mathrm{Mean}\;\bar{x}$	Std.		$\mathrm{Mean}\;\bar{x}$	CV
Section A	0.593	0.191	0.321	1.633 ++	1.219 +
Section B	0.624	0.244	0.391	1.741 ++	1.625 ++
Section C	0.741	0.432	0.583	2.095 ++	2.456 ++
Section D	0.748	0.293	0.392	2.117 ++	1.631 ++
Section E	0.270	0.048	0.179	0.000	0.000
Section F	0.977	0.469	0.480	2.671 ++	2.053 ++
Section G	0.989	0.416	0.420	2.697 ++	1.776 ++
Section H	1.157	0.652	0.564	3.022 ++	2.388 ++
Section I	1.157	1.108	0.957	3.022 ++	3.487 ++

+: QD1 ≥ 1.5 (moderate stress deviation from reference region). ++: QD1 ≥ 2.0 (severe stress deviation from reference region).

**Table 4:** The stress amplitude level division of IVD used in Figure 10.

Stress Amplitude (MPa)	Level	Stress Amplitude (MPa)	Level
1.1~1.2	12	0.5~0.6	6
1.0~1.1	11	0.4~0.5	5
0.9~1.0	10	0.3~0.4	4
0.8~0.9	9	0.2~0.3	3
0.7~0.8	8	0.1~0.2	2
0.6~0.7	7	0.0~0.1	1

## Data Availability

The original contributions presented in this study are included in the article. Further inquiries can be directed at the corresponding authors.
